# Artificial Membrana Tympani

**Published:** 1853-11

**Authors:** Joseph Toynbee


*Artificial Membrana Tympani.*

[To the Editor of the Medical Times and Gazette.]

SIR,—Allow me, through the medium of your Journal, to suggest to those medical men who are using the artificial membrana tympani, that when it is not in use it should be kept in water. By this means it retains its form, and it is kept quite soft. As at present manufactured by Weiss, the artificial membrana tympani is made of vulcanized India-rubber, not much thicker than ordinary writing paper; I find this material preferable to gutta percha, which is apt to wrinkle.

I am, &c., JOSEPH TOYNBEE.

